# Short-form quality care questionnaire-palliative care has acceptable measurement properties in Brazilian cancer patients

**DOI:** 10.1186/s12904-021-00745-y

**Published:** 2021-03-25

**Authors:** Vinício dos Santos Barros, Daniela Bassi-Dibai, André Pontes-Silva, Laíla Silva Linhares Barros, Adriana Sousa Rêgo, Cid André Fidelis-de-Paula-Gomes, Almir Vieira Dibai-Filho

**Affiliations:** 1 Postgraduate Program in Physical Education, Universidade Federal do Maranhão, São Luís, MA Brazil; 2 Postgraduate Program in Programs Management and Health Services, Universidade Ceuma, São Luís, MA Brazil; 3 Postgraduate Program in Adult Health, Universidade Federal do Maranhão, São Luís, MA Brazil; 4 Presidente Dutra Universitary Hospital, Universidade Federal do Maranhão, São Luís, MA Brazil; 5 Postgraduate Program in Rehabilitation Sciences, Universidade Nove de Julho, São Paulo, SP Brazil; 6 Programa de Pós-Graduação em Educação Física, Universidade Federal do Maranhão, Avenida dos Portugueses, 1966, Núcleo de Esportes, 1° andar, Vila Bacanga, São Luís, MA CEP 65080805 Brazil

**Keywords:** Palliative care, Surveys and questionnaires, Quality of health care

## Abstract

**Background:**

Our objective was to perform the translation, cross-cultural adaptation, and validation of the Quality Care Questionnaire-Palliative Care (QCQ-PC) into Brazilian Portuguese for cancer patients in palliative care. The translation and cross-cultural adaptation comprised the following stages: translation, synthesis of translations, back-translation, analysis by a committee of experts, testing of the pre-final version, and definition of the final version. The evaluated measurement properties were: structural validity using factor analysis, test–retest reliability using the intraclass correlation coefficient (ICC), internal consistency using Cronbach’s alpha, and construct validity using the correlations between the QCQ-PC and other questionnaires already validated in Brazil.

**Results:**

Two hundred and twenty-five cancer patients were included for validity analyses, and a subsample of 30 patients was used for test–retest reliability. The most adequate fit indexes were for the short version of the QCQ-PC (SF-QCQ-PC), with two domains and 12 items. There was adequate reliability and internal consistency, with values of the ICC ≥ 0.83 and Cronbach’s alpha ≥0.82. There were correlations > 0.30 between the SF-QCQ-PC and the Karnofsky Performance Scale, the Palliative Prognostic Index, the sadness domain of the Edmonton Symptom Assessment System, the Barthel Index, and all domains related to the McGill Quality of Life Questionnaire and the European Organization for Research in the Treatment of Cancer Questionnaire-core.

**Conclusion:**

The short version of the SF-QCQ-PC has acceptable psychometric properties for use in Brazil.

**Supplementary Information:**

The online version contains supplementary material available at 10.1186/s12904-021-00745-y.

## Introduction

Palliative care is a form of assistance in the health care area and is increasingly gaining space. Palliative care provides quality of life to patients and their families and reduces the suffering of those who are terminally ill, focusing on the treatment of pain and physical, psychosocial and spiritual problems [1]. It is estimated that, worldwide, only 8% of people in need of palliative care have access to this type of health care. Countries are classified into four groups according to the level of development in palliative care: level 1, no activity detected; level 2, in training; level 3a, isolated provision; level 3b, generalized provision; level 4a, preliminary integration; and level 4b, advanced integration. Based on this classification, Brazil is included in level 3a [2].

There are currently some validated instruments to assess the quality of life of Brazilians in palliative care, such as the European Organization for Research and Treatment of Cancer (EORTC) [3] and the McGill Quality of Life Questionnaire (MQOL) [4]. However, to date, no study in Brazil has evaluated the quality of care reported by the cancer patient in palliative care. Few self-report assessment tools focusing on the quality of health care for this population have been developed worldwide [5]. Among these, a recently developed instrument called the Quality Care Questionnaire-Palliative Care (QCQ-PC) was published and made available for use [6]. This questionnaire comprises 32 items in its original version, with four response options to each item. The QCQ-PC consists of four domains or subscales: (1) adequate communication with health professionals, (2) discussion about the value of life and the objectives of care, (3) support and guidance for comprehensive care needs, and (4) accessibility and sustainability of care.

The original version of the QCQ-PC presented high internal consistency, and good convergent and discriminant validity [6]. In addition, this questionnaire has characteristics that highlight it as an important evaluative tool in palliative care, such as the scope of the domains, the presence of items related to the spiritual, social and cultural aspects, and the assessment centered on the self-report of the patient in palliative care. Considering these aspects and the absence of validated instruments to assess the quality of care in palliative care in Brazil, this study aimed to carry out the translation, cross-cultural adaptation, and validation into Brazilian Portuguese of the QCQ-PC in cancer patients in palliative care.

## Methods

### Study design

This is a study of translation, cross-cultural adaptation, and validation of a questionnaire carried out according to the COnsensus-based Standards for the selection of health Measurement INstruments (COSMIN) [7, 8] and Guidelines for the Process of Cross-Cultural Adaptation of Self-Report Measures [9].

The authorization to carry out the translation and validation of the QCQ-PC into Brazilian Portuguese was granted via email by one of the authors of the questionnaire (Dr. Young Ho Yun). The study was carried out in three phases: (1) translation and cross-cultural adaptation of the questionnaire, (2) test of the pre-final version of the translated version of the QCQ-PC into Brazilian Portuguese, and (3) validation of the final version of the QCQ-PC cross-culturally adapted to Brazilian Portuguese.

The research was conducted in the Pain and Palliative Care sector of the Maranhão Cancer Hospital (São Luís, MA, Brazil). This study was carried out in accordance with relevant international guidelines and regulations and the methods was approved by the Research Ethics Committee of the Federal University of Maranhão (opinion number 2.984.884). All participants were 18 years of age or older and signed a free and informed consent form.

### Translation and cross-cultural adaptation

The process of translation and cross-cultural adaptation of the QCQ-PC into Brazilian Portuguese was carried out in six stages, as described below.

1. Translation: Two Brazilians fluent in the English language translated the original version of the QCQ-PC into Brazilian Portuguese.
2. Synthesis of the translations: After discussions and reviews, the two translators, under observation of one of the researchers, produced a single version of the QCQ-PC via consensus.
3. Back-translation: Two English speakers fluent in Portuguese translated the Brazilian Portuguese version of the QCQ-PC back into English, without any prior knowledge of the original version of the questionnaire;
4. Analysis by a committee of experts: Four specialists in the field of palliative care and rehabilitation, together with the four translators involved in the adaptation process, defined the pre-final version of the QCQ-PC in a manner agreed upon by all members of the committee.
5. Pre-final version test: The pre-final version of the QCQ-PC was applied to 30 cancer patients with Brazilian Portuguese as their mother tongue. The patients established their understanding of the pre-final version of the QCQ-PC by checking a checkbox containing the answers “yes” and “no” for each item of the questionnaire. To be considered to have an adequate degree of understanding, items must be understood by at least 80% of participants [10].
6. After analyzing the pre-final version, the coordinator of the cross-cultural adaptation process thus established the final version of the QCQ-PC in Brazilian Portuguese.

### Participants

To calculate the sample size, the rule of seven times the number of items in the questionnaire was used [8]. Considering that the QCQ-PC has 32 items, the minimum adequate sample was established as 224 patients. The eligibility criteria were: ≥ 18 years old; both sexes; cancer diagnosis confirmed by biopsy; ability to read and understand Brazilian Portuguese; no diagnosed cognitive changes; and awareness of the cancer diagnosis.

### QCQ-PC

The original version of the QCQ-PC comprises 32 items, each with four options, namely 1 (strongly agree), 2 (agree), 3 (disagree), and 4 (strongly disagree). The original version of the QCQ-PC consists of four subscales: (1) adequate communication with health professionals; (2) discussion about the value of life and the objectives of care; (3) support and guidance for comprehensive care needs; and (4) accessibility and sustainability of care. The minimum score is 0 and the maximum is 100 points. The higher the score, the better the satisfaction with the quality of care provided by the health team [6].

To calculate the score of the four subscales, the following equations were performed. Subscale 1 score: add the score of items 1–10, subtract 10 from that value, and divide by 0.30. Subscale 2 score: add the score of items 11–19, subtract 9 from that value, and divide by 0.27. Subscale 3 score: add the score of items 20–26, subtract 7 from that value, and divide by 0.21. Subscale 4 score: add the score of items 27–32, subtract 6 from that value, and divide by 0.18.

### Other clinical measures

To determine construct validity by means of correlations, patients were asked to complete questionnaires already validated for Brazilian Portuguese. There is no validated questionnaire in Brazilian Portuguese to measure the quality of care in cancer patients. Thus, we use a high number of tools as a way to fully understand the quantity and magnitude of the QCQ-PQ interactions with other constructs.

The European Organization for Research in the Treatment of Cancer Questionnaire-core 30 (EORTC-QLQ-C15-PAL), validated for Brazilian Portuguese [3], aims to measure the quality of life of cancer patients. This instrument consists of 15 items distributed in three subscales or domains, which are: functional scale (five items), symptom scale (nine items), and global health status (one item). The answers to the first 14 questions are given on a 4-point Likert scale, while the last question is evaluated on a 7-point Likert scale. For interpretation, each subscale must be analyzed separately, making it necessary to transform the raw scores into scores ranging from 0 to 100, so that the higher the score for the functional subscale and overall health status, the better the condition of the patient, while a high score for the symptom subscale indicates greater presence of symptoms.

Quality of life was assessed using the MQOL, validated for Brazilian Portuguese [4]. It consists of 16 questions that include five subscales: physical well-being, psychological well-being, existential well-being, support, and physical symptoms. The MQOL uses an 11-point scale, from 0 to 10, so that the score for each domain corresponds to the average of each subscale. The patient’s quality of life is classified so that the worst possible is 0 and the best possible is 10.

Functional independence was assessed using the Barthel Index, validated for Brazilian Portuguese [11]. The index analyzes 10 activities: food, transfers, personal hygiene, use of the bathroom, bathing, walking on a flat surface, walking up and down stairs, dressing, and undressing. The result follows an increasing scale that ranges from 0 to 100 points: The higher the score, the greater the functional independence.

The Edmonton Symptom Assessment System (ESAS) was used to assess and monitor the symptoms of patients in palliative care. It evaluates nine physical and psychological symptoms on a scale from 0 to 10, where 0 represents the absence of symptoms and 10 represents the symptom in its strongest manifestation. The scale can be filled out by a health professional, the patient, or a family member [12].

Functional capacity was assessed using the Karnofsky Performance Scale (KPS) [13]. The scale is scored from 0 to 100%: 100%, normal, without complaints, without signs of illness; 90%, capable of normal activity, few signs or symptoms of illness; 80%, normal activity with some difficulty, some signs and symptoms; 70%, able to take care of themselves, incapable of normal activity or work; 60%, need for some help, able to take care of most of their own needs; 50%, often needs help, needs frequent medical attention; 40%, incapable, needs special care and help; 30%, severely incapacitated, hospital admission indicated but without risk of death; 20%, very sick, need for immediate admission and support or treatment measures; 10%, dying, rapid progression to fatal disease; and 0%, death.

The Palliative Prognostic Index (PPI) was used to verify the patients’ survival estimate. The scale was developed to predict survival in patients with terminal cancer. The PPI includes the following variables: the KPS, oral intake, edema, dyspnea at rest, and delirium. Based on the impact of each variable to predict patient survival, a final score is created with the sum of scores for each variable. The total score ranges from 0 to 15, and it is possible to stratify patients into three groups with different survival probabilities: PPI ≤ 4 (> 20% chance of survival over the next 6 weeks), 4 < PPI < 6 (20% chance of survival next 6 weeks), and PPI ≥ 6 (20% survival over the next 3 weeks) [14].

### Statistical analysis

Sociodemographic and clinical data are presented as the mean and standard deviation (SD) (quantitative data) or as the absolute number and percent (qualitative data). Internal consistency was calculated using Cronbach’s alpha, considering values between 0.70 and 0.95 to indicate good internal consistency [15].

Reliability was assessed based on a test–retest model, with an interval of 3 to 7 days between assessments. The intraclass correlation coefficient (ICC_2,1_), 95% confidence interval (CI), standard error of measurement (SEM), and minimum detectable difference (MDD) were used to assess the reliability of the total score of each domain of the QCQ-PC [16]. To analyze the reliability of each item in the questionnaire, the kappa coefficient with linear weighting and its respective 95% CI was used.

For the interpretation of the ICC value, the Fleiss study classification was used [17]: For values below 0.40, the reliability was considered low; between 0.40 and 0.75, moderate; between 0.75 and 0.90, substantial; and greater than 0.90, excellent. For kappa interpretation, the following classification was used: < 0, poor; 0.01–0.20, light; 0.21–0.40, reasonable; 0.41–0.60, moderate; 0.61–0.80, substantial; and 0.81–1.00, almost perfect [18].

For the correlations between the questionnaires, the normality of the data was initially verified using the Kolmogorov–Smirnov test. To determine construct validity, Spearman’s correlation coefficient (r_s_) was used to determine the magnitude of the correlation between the QCQ-PC and other measurement instruments: the EORTC-QLQ-C15-PAL, the MQOL, the Barthel Index, the ESAS, the KPS, and the PPI. The interpretation of the magnitude of the correlations used the following criteria: correlations with instruments that measure similar constructs must be ≥0.50; correlations with instruments that measure related but different constructs should be 0.30–0.50; and correlations with instruments that measure unrelated constructs must be < 0.30 [8]. Our hypothesis is that the QCQ-PC presents a correlation with a magnitude between 0.30–0.50 with the measures of quality of life and functional capacity, and a correlation < 0.30 with the symptoms related to cancer.

Floor and ceiling effects were assessed in the present study. By definition, these effects occur when a number of study participants (more than 15%) reach the minimum or maximum value of the questionnaire, which indicates a problem in the instrument’s responsiveness and in the ability of the instrument to differentiate between respondents who have values below the lowest level measured or above the highest level measured.

Internal consistency, reliability and correlations were processed using SPSS version 17.0 (SPSS Inc., Chicago, IL, USA); a 5% significance level was adopted.

For structural validity, exploratory factor analysis (EFA) was initially used with the implementation of a polychoric matrix and a robust diagonally weighted least squares extraction method (RDWLS), because the response possibilities for each item of the QCQ-PC are ordinal values [19, 20]. The identification of the number of factors to be retained was defined by means of parallel analysis with random permutation of the observed data; the rotation was the robust promin [21, 22].

Data processing was performed using the FACTOR software (Universitat Rovira i Virgili, Tarragona, Spain). The adequacy of the model was assessed using the Kaiser–Meyer–Olkin (KMO) criterion and Bartlett’s sphericity test. A KMO value > 0.70 and significant *p* value in the Bartlett test were considered adequate fit indexes [23, 24].

Confirmatory factor analysis (CFA) was performed with the R Studio software (Boston, MA, USA), using the lavaan and semPlot packages. CFA was performed with the implementation of a polychoric matrix and the RDWLS extraction method. The model fit was evaluated by the following indexes: root mean square error of approximation (RMSEA) with 90% CI, comparative fit index (CFI), Tucker–Lewis index (TLI), standardized root mean square residual (SRMR), and chi-square/degrees of freedom (DF). In the present study, values > 0.90 were considered adequate for CFI and TLI, and values < 0.08 were considered adequate for RMSEA and SRMR. Values < 3.00 were considered adequate when interpreting the chi-square/DF [25, 26].

In CFA, factor loadings ≥0.40 were considered adequate for the domain. For comparison between the QCQ-PC models, that is, the original version of the questionnaire versus the versions proposed in this study, the following indexes were used: Akaike information criterion (AIC) and Bayesian information criterion (BIC). The model with the lowest AIC and BIC values was considered the most appropriate model.

## Results

### Cross-cultural adaptation

The following adaptations were made to the QCQ-PC, based on suggestions made by the expert committee, to facilitate the understanding of the questionnaire: (1) the term “medical staff” was adapted to “*equipe de saúde*” (“health staff”) in the instructions for filling out the questionnaire and in items 1–3, 5, 7, 9–12, 15, 17, 19, 21–24, 26, and 30; (2) the term “care plan” was adapted to “*plano de tratamento*” (“treatment plan”) in items 7, 8, 14, 17, 18, and 31; and (3) the term “and” was adapted to “*e/ou*” (“and/or”) in items 20 (“I was able to receive outpatient care and/or telephone counseling with plenty of time) and 25 (“Outpatient care and/or telephone counseling were done at the appointed time without delay”). The use of the term “and/or” was recommended to facilitate understanding and adapt the meaning of the sentence to the different clinical realities, since some patients received only outpatient care, without telephone contacts.

After these adaptations, the pre-final version of the QCQ-PC was defined and applied to 30 patients in palliative care with Brazilian Portuguese as their mother tongue; there was 100% understanding. In this way, the final version of the QCQ-PC in the Brazilian Portuguese language with 32 items was established.

### Sample characteristics

Data collection started in October 2018 and ended in December 2019. Two hundred and twenty-five cancer patients were included for the analysis of floor and ceiling effects, construct validity, and structural validity. The average age of these patients was 55.73 (SD = 15.14) years and the average treatment time was 9.87 (SD = 9.43) months. Twenty-three patients were excluded due to death and 14 due to a lower level of consciousness during the test–retest phase. A total of 30 patients completed the test–retest phase (reliability and internal consistency). The average age of this subsample was 51.06 (SD = 15.64) years and average treatment time was 12.26 (SD = 11.77) months. Table 1 shows the other personal and clinical characteristics of the study participants.

**Table 1:** Descriptive analysis of patients’ personal and clinical characteristics according to the study phases

Variables	Subsample (***n*** = 30)	Sample (***n*** = 225)
Sex		
Male	12 (40)	113 (50.2)
Female	18 (60)	112 (49.8)
Marital status		
Single	9 (30)	50 (22.2)
Married	18 (60)	119 (52.9)
Widower	3 (10)	34 (15.1)
Divorced	0 (0)	22 (9.8)
Educational level		
Elementary	9 (30)	72 (32)
Basic	11 (36.7)	80 (35.6)
High school	8 (26.7)	66 (29.3)
Higher education	2 (6.7)	7 (3.1)
Professional activity		
Active	24 (80)	144 (64)
Inactive	6 (20)	81 (36)
Cancer: primary site		
Uterus	8 (26.7)	39 (17.3)
Stomach	3 (10)	34 (15.1)
Liver	6 (20)	20 (8.9)
Lung	3 (10)	26 (11.6)
Prostate	1 (3.3)	20 (8.9)
Leukemia	2 (6.7)	15 (6.7)
Breast	1 (3.3)	10 (4.4)
Lymphoma	0 (0)	6 (2.7)
Kidney	3 (10)	9 (4)
Osteosarcoma	1 (3.3)	5 (2.2)
Esophagus	0 (0)	5 (2.2)
Penis	0 (0)	4 (1.8)
Multiple myeloma	0 (0)	4 (1.8)
Others	2 (6.7)	28 (12.44)
Type of treatment		
Palliative	21 (70)	115 (51.1)
Curative	4 (13.3)	72 (32)
Both	5 (16.7)	38 (16.9)
Current treatment		
Drug therapy	26 (86.7)	122 (54.2)
Chemotherapy	3 (10)	69 (30.7)
Radiotherapy	0 (0)	11 (4.9)
Surgical	1 (3.3)	23 (10.2)
Metastasis		
Yes	15 (50)	129 (57.3)
No	15 (50)	96 (42.7)

Values presented in absolute number (percentage)

Regarding to the other clinical variables, Table 2 presents the scores for the KPS, the PPI, the ESAS, the Barthel Index, the MQOL, the EORTC-QLQ-C15-PAL, and the QCQ-PC.

**Table 2:** Descriptive analysis of clinical evaluations according to the phases of the study

Variables	Subsample (*n* = 30)	Sample (*n* = 225)
KPS (score)	60.00 (12.86)	61.46 (16.47)
PPI (score)	2.96 (1.83)	2.33 (1.89)
ESAS (score)		
Pain	4.83 (3.17)	3.76 (3.13)
Tiredness	4.20 (3.37)	3.32 (2.83)
Nausea	3.06 (3.20)	3.24 (2.99)
Sadness	5.90 (3.57)	5.34 (3.07)
Anxiety	6.83 (3.20)	6.13 (3.14)
Somnolence	4.36 (2.73)	4.04 (2.76)
Appetite	4.26 (2.59)	3.72 (2.48)
Welfare	4.93 (2.27)	3.73 (2.20)
Dyspnea	3.40 (3.73)	1.95 (2.59)
Barthel Index (score)	70.33 (22.39)	72.39 (19.92)
MQOL (score)		
Physical symptoms	3.83 (1.88)	4.90 (4.63)
Physical well-being	4.76 (1.86)	5.55 (2.64)
Psychological	2.85 (1.31)	4.20 (2.18)
Existential	4.71 (1.54)	5.40 (1.91)
Support	6.76 (1.38)	7.05 (1.91)
EORTC-QLQ-C15-PAL (score)		
Functional	60.66 (24.15)	44.44 (25.27)
Symptoms	44.44 (19.98)	36.88 (20.33)
Quality of life	45.00 (25.94)	55.33 (28.80)

Values shown as mean (standard deviation). *KPS* Karnofsky Performance Scale, *PPI* Palliative Prognostic Index, *ESAS* Edmonton Symptom Assessment System, *MQOL* McGill Quality of Life Questionnaire, *EORTC-QLQ-C15-PAL* European Organization for Research in the Treatment of Cancer Questionnaire

### Structural validity

**Fig. 1:**
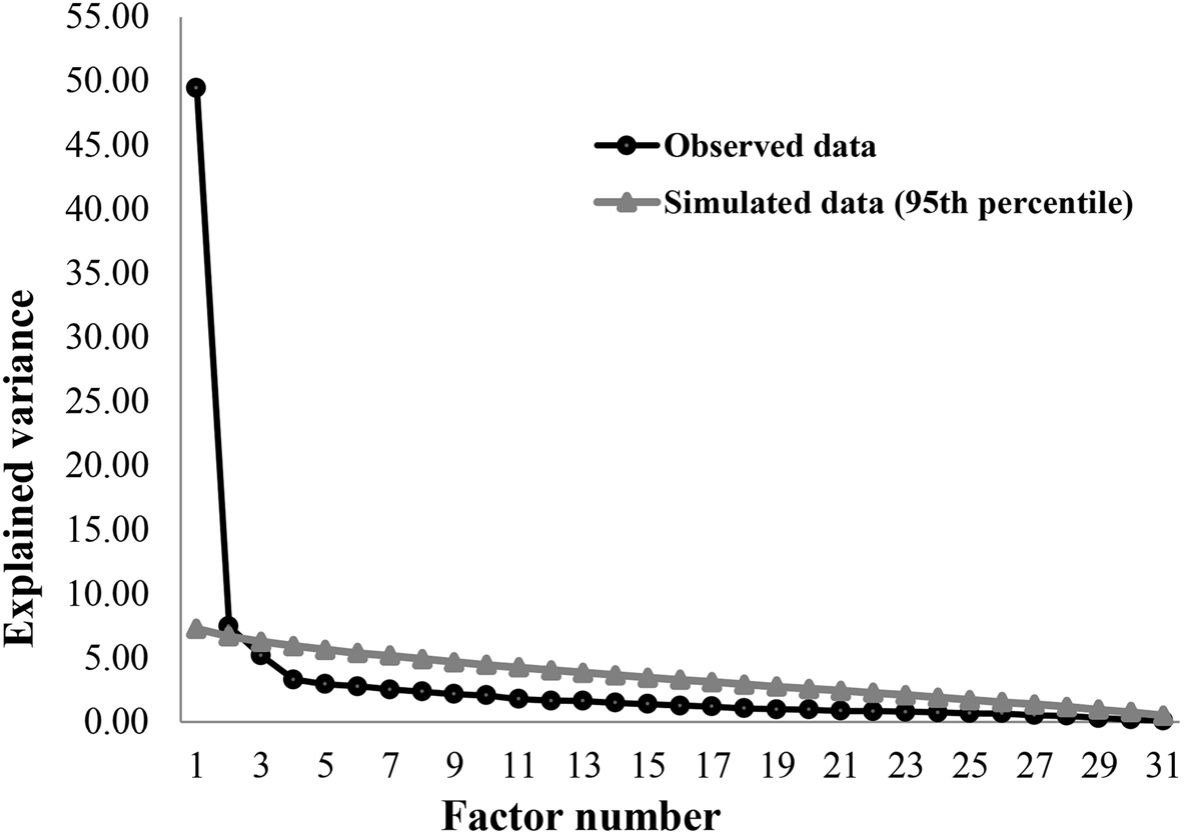
Scree plot with the definition of two factors by the parallel analysis of the Quality Care Questionnaire-Palliative Care (QCQ-PC)

Initially, CFA of the full Brazilian version of the QCQ-PC was performed (four domains and 32 items). However, in view of the inadequate values of some fit indexes, EFA was performed to identify the number of domains according to the parallel analysis in the data of the present study, as shown in Fig. 1. EFA presented adequate fit indexes (KMO = 0.95, *p* < 0.001 for Bartlett’s sphericity test). Thus, for the purpose of comparison between the structures of the QCQ-PC, the full Brazilian version was considered as Model 1, with four domains and 32 items (domain 1 = items 1–9; domain 2 = items 11–19; domain 3 = items 20–26; and domain 4 = items 27–32).

Model 2 was defined after EFA and exclusion of items with factor loading < 0.40 (items 20 and 25), with the QCQ-PC version being defined with two domains and 30 items (domain 1 = items 1–8, 10–13, 15–17, 19, 26, 27, and 30–32; and domain 2 = items 9, 14, 18, 21–24, 28, and 29). However, after analysis of the questionnaire items by the researchers, the two domains identified by the parallel analysis were called “communication with health professionals” and “care and assistance provided by health professionals.” Therefore, through theoretical analysis of the QCQ-PC Model 2, based on these two domains, items with a different meaning from the domain context or redundant were excluded by the qualitative analysis of the researchers in consensus. Consequently, Model 3 was established with two domains and 12 items (domain 1 = items 2, 4, 5, 8, 11, and 16; and domain 2 = items 14, 18, 21–23, and 28). Table 3 presents the items of the QCQ-PC and the exclusions made based on the factor analysis and the qualitative analysis.

**Table 3:** Items of the Quality Care Questionnaire-Palliative Care (QCQ-PC) maintained and excluded in the qualitative analysis carried out by researchers according to the two domains defined in the factor analysis

Item		Decision
**Communication with health professionals**		
1	I am satisfied with the careful manner of medical staff (Eu estou satisfeito(a) com a conduta cuidadosa da equipe de saúde).	A
2	I am satisfied with the way of communication of medical staff (Eu estou satisfeito(a) com a forma com que a equipe de saúde se comunica).	B
3	I was able to receive adequate care from medical staff (Eu recebi cuidados adequados da equipe de saúde).	A
4	I have heard and understood an accurate description of the progress of my disease (Eu ouvi e compreendi a descrição precisa do progresso de minha doença).	B
5	The medical staff explained terms that I was curious about (A equipe de saúde me explicou os termos médicos pelos quais demonstrei curiosidade).	B
6	I was able to receive the healthcare service I demanded (Eu recebi os cuidados em saúde que precisava).	A
7	The medical staff support my decision on care plan (A equipe de saúde apoiou minha decisão sobre o meu plano de tratamento).	A
8	I have heard and understood an accurate description of my care plan (Eu ouvi e compreendi a descrição precisa do meu plano de tratamento).	B
10	The medical staff paid attention to various symptoms I felt and adjusted them well (A equipe de saúde prestou atenção em vários dos meus sintomas e os tratou adequadamente).	A
11	I was able to discourse with medical staff about the value of my life (Eu consegui conversar com a equipe de saúde sobre o valor da minha vida).	B
12	I was able to recall what is important to achieve the values and goals of my life while discoursing with medical staff (Eu consegui lembrar o que é importante para alcançar os valores e objetivos da minha vida enquanto conversava com a equipe de saúde).	A
13	I was able to express what my family and I expected from care (Eu consegui expressar o que minha família e eu esperávamos do tratamento).	A
15	I was able to receive adequate help from medical staff, while I was having difficulties in setting up specific goals related to care (Eu recebi ajuda adequada da equipe de saúde enquanto eu estava tendo dificuldades em estabelecer metas específicas do meu tratamento).	A
16	My family and I received an education that is helpful to care (Minha família e eu recebemos informações úteis sobre o meu tratamento).	B
17	The medical staff suggested an adequate care plan in consideration of values of my life (A equipe de saúde sugeriu um plano de tratamento adequado, considerando os valores da minha vida).	A
19	The medical staff managed intermediate checkups to verify whether I could execute my goals (A equipe de saúde realizou exames intermediários para verificar se eu poderia alcançar meus objetivos).	A
26	My family and I received psychological support from medical staff (Minha família e eu recebemos apoio psicológico da equipe de saúde).	A
27	Services needed for my care are provided by experts in their respective fields (Os atendimentos necessários para o meu tratamento foram realizados por especialistas em suas respectivas áreas).	A
30	The medical staff periodically confirmed my goals and plans toward care (A equipe de saúde confirmou periodicamente os objetivos e planos para o meu tratamento).	A
31	The decision on a healthcare plan was reflected by my family and my opinions (A decisão sobre um plano de tratamento levou em consideração a minha família e as minhas opiniões).	A
32	I understand the goal of care (Eu entendi o objetivo do tratamento).	A
**Care and assistance provided by health professionals**		
9	I was able to have a conversation with medical staff in a relaxed atmosphere (Eu consegui conversar com a equipe de saúde em um ambiente descontraído).	A
14	My care plans included the things I was able to try myself (Meus planos de tratamento incluíam as coisas que eu poderia fazer).	B
18	I was able to modify my plan when my demand for treatment changed (Eu consegui modificar meu plano de tratamento quando minhas necessidades mudaram).	B
21	The medical staff provide support to me and my family to solve spiritual concerns (A equipe de saúde forneceu suporte para mim e minha família resolvermos assuntos espirituais).	B
22	The medical staff provided support to me and my family to overcome social crisis (A equipe de saúde forneceu suporte para mim e minha família resolvermos problemas sociais).	B
23	The medical staff knew what I wanted (A equipe de saúde sabia o que eu queria).	B
24	The medical staff communicated smoothly with me and my family (A equipe de saúde se comunicou sem dificuldades comigo e com minha família).	A
28	I was able to get care services at the locations I wanted (Obtive assistência dos profissionais de saúde nos locais que eu queria).	B
29	Medical care is immediately provided in a state of crisis (Os cuidados em saúde foram realizados imediatamente durante as crises).	A

Items 20 (I was able to receive outpatient care and telephone counseling with plenty of time) and 25 (Outpatient care and telephone counseling were done at the appointed time without delay) were excluded after exploratory factor analysis (factor loading < 0.40). Decision A: Item excluded because it has a different meaning from the domain context or has redundancy; Decision B: Item included in the Short-Form QCQ-PC

**Fig. 2:**
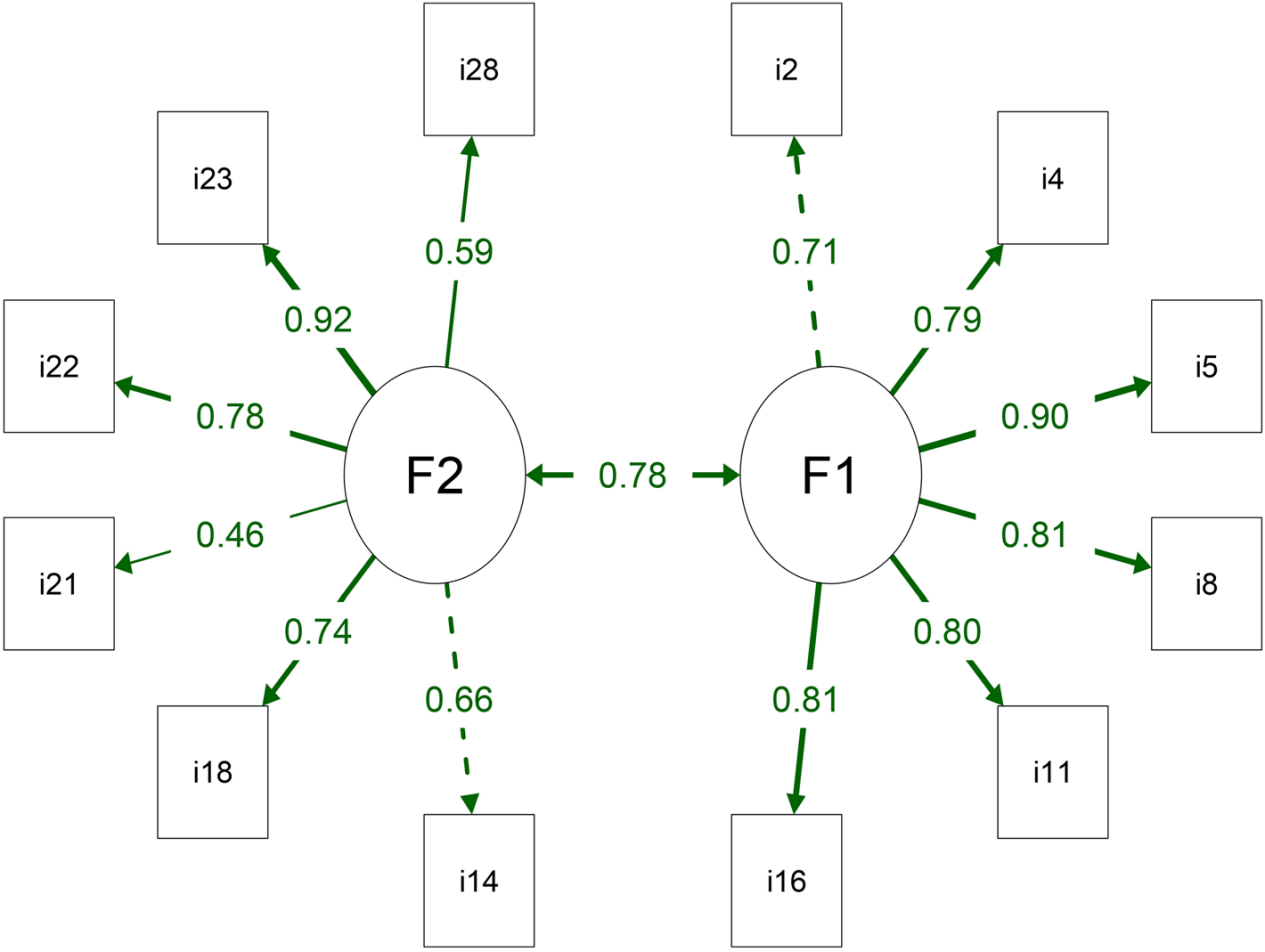
Path diagram with factor loads for each domain of the Short-Form Quality Care Questionnaire-Palliative Care (SF-QCQ-PC). F1: Communication with health professionals; F2: Care and assistance provided by health professionals

Table 4 shows the comparisons between the three models tested here. Model 3 presented more adequate fit indexes, even with lower AIC and BIC values. Figure 2 shows the structure with the Model 3 factor loadings, which varied between 0.46 and 0.92. The Short-Form QCQ-PC (SF-QCQ-PC) with two domains and 12 items in Brazilian Portuguese and English is presented in Additional files 1 and 2, respectively. The following equation was used to calculate the score for each domain: the score of all items in the domain was added, 6 was subtracted from the value, and it was divided by 0.18.

**Table 4:** Confirmatory factor analysis of the three structures of the Quality Care Questionnaire-Palliative Care (QCQ-PC) tested in the present study

Models	Chi-square	DF	Chi-square/DF	CFI	TLI	RMSEA (90% CI)	SRMR	AIC	BIC
Model 1	1474.697	458	3.21	0.954	0.950	0.100 (0.094, 0.105)	0.089	9820.596	10,059.723
Model 2	974.895	404	2.41	0.974	0.972	0.079 (0.073, 0.086)	0.070	8882.716	9091.099
Model 3	104.552	53	1.97	0.987	0.984	0.066 (0.047, 0.079)	0.053	4532.725	4618.128

*DF* Degree of freedom, *CFI* Comparative fit index, *TLI* Tucker-Lewis Index, *RMSEA* Root mean square error of approximation, *CI* Confidence interval, *SRMR* Standardized root mean square residual, *AIC* Akaike information criterion, *BIC* Bayesian information criterion. Model 1: QCQ-PC with 4 domain and 32 items (original version); Model 2: QCQ-PC with 2 domains and 30 items; Model 3: QCQ-PC with 2 domains and 12 items

### Reliability and internal consistency

The kappa coefficient was used to examine the reliability analysis of each item of the SF-QCQ-PC, as shown in Table 5. For domain 1, there were adequate kappa values (between 0.40 and 0.52). For domain 2, the kappa values were also adequate (between 0.43 and 0.66).

**Table 5:** Reliability of each item of the Short-Form Quality Care Questionnaire-Palliative Care (SF-QCQ-PC) according to each domain

SF-QCQ-PC	Mean (standard deviation)		Kappa (95% IC)	Cronbach’s alpha if the item is deleted
	Test	Retest		
Domain 1				
Item 2	3.13 (0.68)	3.33 (0.66)	0.52 (0.27, 0.78)	0.81
Item 4	3.20 (0.66)	3.26 (0.44)	0.50 (0.27, 0.72)	0.84
Item 5	3.23 (0.62)	3.26 (0.58)	0.49 (0.19, 0.79)	0.79
Item 8	3.33 (0.66)	3.23 (0.56)	0.52 (0.24, 0.80)	0.80
Item 11	3.06 (0.69)	3.13 (0.62)	0.40 (0.12, 0.68)	0.81
Item 16	3.53 (0.50)	3.40 (0.49)	0.40 (0.08, 0.72)	0.82
Domain 2				
Item 14	2.76 (0.77)	2.96 (0.71)	0.50 (0.28, 0.72)	0.80
Item 18	2.63 (0.66)	2.73 (0.78)	0.51 (0.31, 0.71)	0.80
Item 21	2.70 (0.98)	2.90 (0.80)	0.59 (0.38, 0.79)	0.82
Item 22	3.53 (0.50)	3.56 (0.56)	0.43 (0.13, 0.72)	0.83
Item 23	3.06 (0.58)	3.13 (0.62)	0.66 (0.41, 0.91)	0.81
Item 28	2.60 (0.85)	2.70 (0.87)	0.53 (0.27, 0.78)	0.82

*CI* Confidence interval. Domain 1: Communication with health professionals; Domain 2: Care and assistance provided by the health professionals

There was adequate reliability for the domains 1 and 2 total scores of the SF-QCQ-PC, with ICC ≥ 0.83 and SEM ≤ 6.81%, as shown in Table 6. There was also adequate internal consistency, with Cronbach’s alpha ≥0.82.

**Table 6:** Mean and standard deviation of the test and retest, reliability of the score by domain and internal consistency of the Shor-Form Quality Care Questionnaire-Palliative Care (*n* = 30)

Domains	Test	Retest	ICC (95% CI)	SEM (score)	SEM (%)	MDC (score)	MDC (%)	Cronbach’s alpha
Domain 1	74.99 (13.43)	75.74 (12.24)	0.83 (0.64, 0.92)	5.13	6.81	14.23	18.88	0.82
Domain 2	62.77 (15.58)	66.66 (13.73)	0.92 (0.83, 0.96)	4.15	6.21	11.49	17.22	0.83

Domain 1: Communication with health professionals; Domain 2: Care and assistance provided by the health professionals; *ICC* Intraclass correlation coefficient, *CI* Confidence interval, *SEM* Standard error of measurement, *MDC* Minimal detectable change

### Construct validity: correlation between questionnaires

Considering that no tool used in the current study presents a construct similar to satisfaction with care and assistance, correlations > 0.30 are considered to be satisfactory. As shown in Table 7, there were correlations > 0.3 between domain 1 of the SF-QCQ-PC and the KPS, the PPI, the sadness domain of the ESAS, the Barthel Index, and all domains related to the quality of life of the QQVM and the EORTC-QLQ -C15-PAL. There were also correlations > 0.3 between domain 2 of the SF-QCQ-PC and the KPS, the PPI, the Barthel Index, and all domains related to the quality of life of the MQOL and the EORTC-QLQ-C15-PAL.

**Table 7:** Correlation between the score of the Short-Form Quality Care Questionnaire-Palliative Care domains and the other study variables (*n* = 225)

Variable	SF-QCQ-PC	
	Domain 1	Domain 2
KPS (score)	r_s_ = 0.550*	r_s_ = 0.491*
PPI (score)	r_s_ = − 0.523*	r_s_ = − 0.354*
ESAS (score)		
Pain	r_s_ = − 0.243*	r_s_ = − 0.183*
Tiredness	r_s_ = − 0.258*	r_s_ = − 0.212*
Nausea	r_s_ = − 0.249*	r_s_ = − 0.250*
Sadness	r_s_ = − 0.306*	r_s_ = − 0.146*
Anxiety	r_s_ = − 0.260*	r_s_ = − 0.093
Somnolence	r_s_ = − 0.232*	r_s_ = − 0.167*
Appetite	r_s_ = − 0.180*	r_s_ = − 0.086
Welfare	r_s_ = − 0.214*	r_s_ = − 0.081
Dyspnea	r_s_ = − 0.177*	r_s_ = − 0.039
Barthel Index (score)	r_s_ = 0.450*	r_s_ = 0.500*
MQOL (score)		
Physical symptoms	r_s_ = 0.557*	r_s_ = 0.421*
Physical well-being	r_s_ = 0.565*	r_s_ = 0.496*
Psychological	r_s_ = 0.466*	r_s_ = 0.468*
Existential	r_s_ = 0.644*	r_s_ = 0.558*
Support	r_s_ = 0.682*	r_s_ = 0.460*
EORTC-QLQ-C15-PAL (score)		
Functional	r_s_ = − 0.491*	r_s_ = − 0.362*
Symptoms	r_s_ = − 0.437*	r_s_ = − 0.382*
Quality of life	r_s_ = 0.507*	r_s_ = 0.457*

Domain 1: Communication with health professionals; Domain 2: Care and assistance provided by the health professionals. *KPS* Karnofsky Performance Scale, *PPI* Palliative Prognostic Index, *ESAS* Edmonton Symptom Assessment System, *MQOL* McGill Quality of Life Questionnaire, *EORTC-QLQ-C15-PAL* European Organization for Research in the Treatment of Cancer Questionnaire. *Significant correlation (*p* < 0.05, Spearman’s correlation coefficient)

### Floor and ceiling effects

With respect to domains 1 and 2, no patient reached the minimum score (0). With respect to the maximum score, 25 (11.1%) patients reached 100 points in domain 1 and 2 (0.9%) patients reached 100 points in the domain 2. Thus, there were no floor and ceiling effects in the SF-QCQ-PC.

## Discussion

The results of the present study show that the Brazilian version of the SF-QCQ-PC has a more adequate structure with two domains and 12 items, contrasting the original model with four domains and 32 items. In addition, the SF-QCQ-PC has adequate reliability, internal consistency, and construct validity, with good applicability and understanding. There were no floor and ceiling effects.

In the SF-QCQ-PC, there are two subscales: (1) communication with health professionals and (2) care and assistance provided by health professionals. The original version has four subscales: (1) adequate communication with health professionals; (2) discussion about the value of life and the objectives of care; (3) support and guidance for comprehensive care needs; and (4) accessibility and sustainability of care [6]. An important difference between the original version of the QCQ-PC and our study concerns the methodology used for structural validity. In the original version, the authors performed EFA without describing which method they used to identify the four domains [6]. The present study used EFA with the implementation of parallel analysis to identify the two domains. Parallel analysis is currently considered the most reliable method for performing EFA because it overcomes the gaps of other methods that use an eigenvalue > 1 and inflection of the scree plot, which tend to overestimate the number of domains [27].

The Brazilian version of the SF-QCQ-PC showed an adequate correlation with related constructs, that is, quality of life, symptoms related to cancer, functional independence, and estimated survival. Regarding construct validity through correlations between instruments, the authors of the original version of the QCQ-PC [6] reported fewer correlations and lower correlation magnitudes than the present study, with emphasis on the EORTC-QLQ-C15-PAL and the MQOL. The original version was not correlated with the functional domain of the EORTC-QLQ-C15-PAL. In the present study, there was a correlation between the SF-QCQ-PC and the functional domain of the EORTC-QLQ-C15-PAL. In addition to patients showing good understanding and adequate psychometric properties, the version in the present study is short and, consequently, requires less time for the patient to complete it.

Another highlight of the present study was the reliability (test–retest). Reliability is associated with score consistency, which refers to the proportion of total variance of the measure that is due to the true differences among patients [8]. There was adequate reliability for the total score of domains 1 and 2 of the SF-QCQ-PC, with ICC values of 0.83 and 0.92, respectively. ICC values > 0.75 are suggested for health measurement instruments [28]. The original version describes as a limitation the failure to perform reliability analysis (test–retest).

In Brazilian Portuguese, some questionnaires have been developed to assess patient satisfaction with services in the field of physiotherapy, such as the quality of care provided in physiotherapy sessions [29, 30] and in outpatient physiotherapy [31]. However, our study is pioneering in validating a questionnaire to assess the quality of specific care for cancer patients in palliative care.

With regard to the clinical applicability, the SF-QCQ-PC has positive points for assessing the quality of care for cancer patients, such as: the small number of items; the short time to answer the questionnaire; the assessment of two crucial aspects in the clinical care of these patients (communication and assistance); the presence of items related to spiritual and social aspects; and the presence of items that allow the assessment of the care received by the entire health team (not being restricted to certain professional classes). In this way, Brazilian professionals working in palliative care can use the SF-QCQ-PC as a parameter for implementing measures and monitoring the quality of care provided.

This study, like all, has limitations that must be considered. Despite having been guided by factor analysis, the definition of Model 3 in this study presents a considerable degree of subjectivity due to the exclusion of items based on the qualitative analysis carried out by the researchers. The sample entirely comprised patients seen at the hospital service, not including patients seen at outpatient clinics or at home. In addition, patients who were in a serious condition (altered level of consciousness) were not evaluated. This criterion is evaluated daily by the psychology team in the palliative care sector of the service where the study was conducted. The proposed short version of the QCQ-PC presented acceptable measurement properties in Brazilian Portuguese; however, it should be investigated in other languages. To date, only the original version of the QCQ-PC has been published, and there have been no other cross-culturally adapted and validated versions, a fact that limited discussion and comparison with more studies.

## Conclusion

The Brazilian version of the SF-QCQ-PC with two domains and 12 items has acceptable psychometric properties. Thus, its use in cancer patients in palliative care has scientific support.

## Supplementary Information

**Additional file 1:** Short-Form Quality Care Questionnaire-Palliative Care (SF-QCQ-PC) in Brazilian Portuguese.

**Additional file 2:** Short-Form Quality Care Questionnaire-Palliative Care (SF-QCQ-PC) in English.

## Data Availability

Data available in supplementary file.
