# Supplementary material for: Short-form quality care questionnaire-palliative care has acceptable measurement properties in Brazilian cancer patients
Source: BMC Palliat Care. 2021 Mar 25;20:49. doi: 10.1186/s12904-021-00745-y (PMC7993463; doi:10.1186/s12904-021-00745-y)
Supplement: Supplementary file 1 — Additional file 1: Short-Form Quality Care Questionnaire-Palliative Care (SF-QCQ-PC) in Brazilian Portuguese. [file 12904_2021_745_MOESM1_ESM.docx]

**Additional file 1.** Short-Form Quality Care Questionnaire-Palliative Care (SF-QCQ-PC) in Brazilian Portuguese.

**Versão curta do Quality Care Questionnaire-Palliative Care (SF-QCQ-PC)**

Nome ______________________________________________________________________________________________ Data: _______________________

Os itens abaixo estão relacionados à sua opinião sobre os cuidados de saúde fornecidos pela equipe de saúde que acompanha o seu tratamento. Por favor, leia cada item a seguir e assinale o número correspondente à sua opinião.

| Nº | Questões | Concordo totalmente | Concordo | Discordo | Discordo totalmente |
| --- | --- | --- | --- | --- | --- |
| Comunicação com os profissionais de saúde | | | | | |
| 1 | Eu estou satisfeito(a) com a forma com que a equipe de saúde se comunica. | 4 | 3 | 2 | 1 |
| 2 | Eu ouvi e compreendi a descrição precisa do progresso de minha doença. | 4 | 3 | 2 | 1 |
| 3 | A equipe de saúde me explicou os termos médicos pelos quais demonstrei curiosidade. | 4 | 3 | 2 | 1 |
| 4 | Eu ouvi e compreendi a descrição precisa do meu plano de tratamento. | 4 | 3 | 2 | 1 |
| 5 | Eu consegui conversar com a equipe de saúde sobre o valor da minha vida | 4 | 3 | 2 | 1 |
| 6 | Minha família e eu recebemos informações úteis sobre o meu tratamento. | 4 | 3 | 2 | 1 |
| Cuidado e assistência prestados pelos profissionais de saúde | | | | | |
| 7 | Meus planos de tratamento incluíam as coisas que eu poderia fazer. | 4 | 3 | 2 | 1 |
| 8 | Eu consegui modificar meu plano de tratamento quando minhas necessidades mudaram. | 4 | 3 | 2 | 1 |
| 9 | A equipe de saúde forneceu suporte para mim e minha família resolvermos assuntos espirituais. | 4 | 3 | 2 | 1 |
| 10 | A equipe de saúde forneceu suporte para mim e minha família resolvermos problemas sociais. | 4 | 3 | 2 | 1 |
| 11 | A equipe de saúde sabia o que eu queria. | 4 | 3 | 2 | 1 |
| 12 | Obtive assistência dos profissionais de saúde nos locais que eu queria. | 4 | 3 | 2 | 1 |
