# Supplementary material for: Short-form quality care questionnaire-palliative care has acceptable measurement properties in Brazilian cancer patients
Source: BMC Palliat Care. 2021 Mar 25;20:49. doi: 10.1186/s12904-021-00745-y (PMC7993463; doi:10.1186/s12904-021-00745-y)
Supplement: Supplementary file 2 — Additional file 2: Short-Form Quality Care Questionnaire-Palliative Care (SF-QCQ-PC) in English. [file 12904_2021_745_MOESM2_ESM.docx]

**Additional file 2.** Short-Form Quality Care Questionnaire-Palliative Care (SF-QCQ-PC) in English.

**Short-Form Quality Care Questionnaire-Palliative Care (SF-QCQ-PC)**

Name ______________________________________________________________________________________________ Date: _______________________

The items below are related to your opinion about the health care provided by the health team that accompanies your treatment. Please read each item below and mark the number corresponding to your opinion.

| Nº | Questions | Strongly  Agree | Agree | Disagree | Strongly  Disagree |
| --- | --- | --- | --- | --- | --- |
| Communication with health professionals | | | | | |
| 1 | I am satisfied with the way of communication of health staff. | 4 | 3 | 2 | 1 |
| 2 | I have heard and understood an accurate description of the progress of my disease. | 4 | 3 | 2 | 1 |
| 3 | The health staff explained terms that I was curious about. | 4 | 3 | 2 | 1 |
| 4 | I have heard and understood an accurate description of my care plan. | 4 | 3 | 2 | 1 |
| 5 | I was able to discourse with health staff about the value of my life. | 4 | 3 | 2 | 1 |
| 6 | My family and I received an education that is helpful to care. | 4 | 3 | 2 | 1 |
| Care and assistance provided by health professionals | | | | | |
| 7 | My care plans included the things I was able to try myself. | 4 | 3 | 2 | 1 |
| 8 | I was able to modify my care plan when my demand for treatment changed. | 4 | 3 | 2 | 1 |
| 9 | The health staff provide support to me and my family to solve spiritual concerns. | 4 | 3 | 2 | 1 |
| 10 | The health staff provided support to me and my family to overcome social crisis. | 4 | 3 | 2 | 1 |
| 11 | The health staff knew what I wanted. | 4 | 3 | 2 | 1 |
| 12 | I was able to get care services at the locations I wanted. | 4 | 3 | 2 | 1 |
